# Diagnostic value of high-risk HPV E6/E7 mRNA in patients with ASCUS

**DOI:** 10.1186/s12905-023-02599-3

**Published:** 2023-09-14

**Authors:** Xiu Jin, Feifei Liu, Ya Zhang, Yingying Ma, Linqing Yang, Yunfei Wang, Ying Liu

**Affiliations:** 1 Department of Pathology, Affiliated Hospital of Jining Medical University, JiNing, ShanDong China; 2 Department of Obstetrics, Affiliated Hospital of Jining Medical University, JiNing, ShanDong China; 3 Department of Gynecology, Affiliated Hospital of Jining Medical University, JiNing, ShanDong China

**Keywords:** Cervical cancer, ASCUS, HPV E6/E7 mRNA, Menopause, Colposcope

## Abstract

**Objective:**

To investigate the infection status of high-risk human papillomavirus (HR-HPV) E6/E7 mRNA in patients with a cytological diagnosis of “atypical squamous cells of undetermined significance” (ASCUS) and to analyze the pathogenic rate of different high-risk HPV subtypes combined with biopsy pathological results to provide a more accurate basis for managing ASCUS patients.

**Methods:**

A total of 1387 patients with ASCUS and HPV E6/E7 mRNA positivity who were referred for colposcopy were retrospectively analyzed. They were divided into HPV16+, 18/45 + and other HR-HPV + groups premenopausal and postmenopausal groups. The pathological results of the biopsy were divided into the LSIL- group (including normal and low-grade squamous intraepithelial lesions) and the HSIL + group (including high-grade squamous intraepithelial lesions and higher lesions). SPSS was used for the analysis.

**Results:**

The age group 31–40 years had the highest level of HPV16+, and HPV18/45 + was the highest in the 41–50 years group. The detection rates of HSIL + in the HPV16+, HPV18/45+, HPV 16/18/45 + and Other HR-HPV + groups were 48.4%, 18.8%, 43.9% and 15.0%, respectively. The infection rates of HPV16/18/45 in postmenopausal and premenopausal women were 42.4% and 34.3%, respectively. In the HPV18/45 group, the incidence of HSIL + was 30.0% in postmenopausal women and 15.0% in premenopausal women (P < 0.01). In the HPV 16 + and Other HR-HPV + groups, the incidence of HSIL + in postmenopausal patients was not significantly different from that in premenopausal patients. The incidence of cervical cancer in postmenopausal patients is significantly higher than that in premenopausal patients.

**Conclusions:**

Colposcopy referral or further biopsy is recommended for all ASCUS patients with HPV16/18/45E6/E7 mRNA positivity and postmenopausal patients with HR-HPVE6/E7 mRNA positivity. For premenopausal ASCUS patients with other HR-HPV E6/E7 mRNA positivity, colposcopy should be performed if possible, depending on the specific situation, to achieve early detection and diagnosis.

## Background

Cervical cancer is one of the most common malignant tumors in women. There are approximately 100,000 new cases of cervical cancer in China every year [1]. The mortality rate of cervical cancer has surpassed that of ovarian cancer and it has become the malignant tumor with the highest mortality rate among gynecological tumors [2]. With the improvement of people’s health awareness, an increasing number of cervical epithelial lesions are found by cervical liquid-based thin layer cytology (TCT). As an uncertain diagnosis, ASCUS has a high detection rate in the Bethesda (TBS) system, which leads to the low accuracy of TCT screening [3]. In 2013, ASCCP recommended the HPV DNA test as a basis for the management of patients with ASCUS, but its specificity was low, and the false-positive rate was high. It was later reported that, compared with HPV DNA detection, HPV E6/E7 mRNA detection has high sensitivity and good specificity in high-grade cervical precancerous lesions, and it can be used as an effective means of further diagnosing patients with ASCUS [4–6].

Persistent infection with high-risk human papillomavirus (HR-HPV) is the main cause of cervical cancer [7]. Among them, HPVl6 and 18 types are the most common in the clinic, and patients with HPVl6/18 coinfection are more likely to develop high-grade lesions (CINII +) [8]. A total of 81.8% of CIN II + patients are HPV16 positive, and 18.1% of patients are HPV18, 31, 33, or 45 subtype positive [9]. It is suggested that the screening and testing of HPV DNA should pay attention to these HPV types [10]. However, in addition to subtypes 16 and 18, it is controversial whether the increasing number of patients positive for other subtypes should be referred for colposcopy and cervical biopsy. Moreover, there is still a lack of clinical management standards and guidance for HPV E6/E7 mRNA-positive ASCUS patients. Therefore, the purpose of this study was to explore the different morbidities of different high-risk subtypes of HPV in patients with ASCUS by analyzing the relationship between the pathological results of biopsy and the results of tests for HPV16, 18/45 and other high-risk subtypes of HPV E6/E7 mRNA in patients with ASCUS to screen for cervical cancer more accurately.

Studies have reported that 20% of all new cases of cervical cancer occur in women aged 65 and above, who account for 34% of cervical cancer deaths [11]. For postmenopausal women, the HR-HPV test can better predict high-grade cervical lesions than TCT [12]. Regardless of whether the previous screening is negative or positive, close attention should be given to cervical cancer screening in postmenopausal women [13]. At present, there is no difference in the recommendations for cervical cancer screening between premenopausal and postmenopausal women. Few studies have reported HPV mRNA infection and corresponding cervical morbidity in premenopausal and postmenopausal ASCUS patients, so we need more clinical and pathological data on premenopausal and postmenopausal ASCUS women. In this study, premenopausal and postmenopausal women were divided into two groups to analyze the relationship between HPV16, 18/45 and Other HR-HPV infection and the pathological results of biopsy to explore the necessity of further colposcopy referral and biopsy for ASCUS patients. The results of this study will provide a basis for pathologists to improve the diagnosis of ASCUS and to provide feasible suggestions for clinicians to make accurate diagnoses and provide appropriate treatments for patients.

## Materials and methods

### General data

**Fig. 1:**
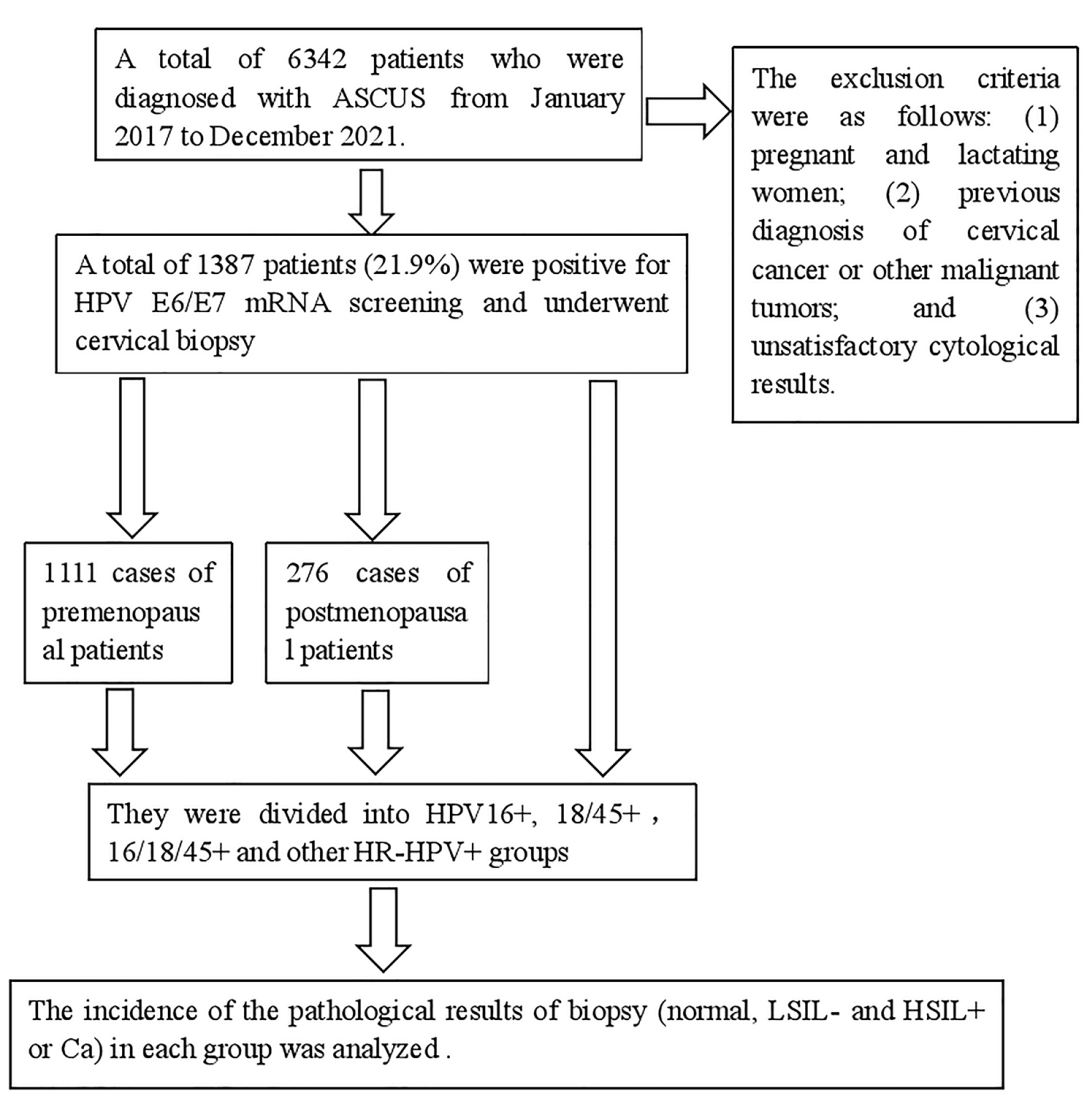
Main flow diagram

A total of 6342 patients who were diagnosed with “atypical squamous cells-unclear meaning” (ASC-US) in the affiliated Hospital of Jining Medical College from January 2017 to December 2021 were selected. A total of 1387 patients (21.9%) with positive HPV E6 /E7 mRNA screening and cervical biopsy were enrolled in this retrospective study.The exclusion criteria were as follows: (1) pregnant and lactating women; (2) previous diagnosis of cervical cancer or other malignant tumors; and (3) unsatisfactory cytological results. The age of the patients ranged from 16 to 77 years old, with an average age of 40.80 ± 11.32 years. The patients positive for the HPV16 or 18/45 subtypes were classified as the HPV16/18/45 subtype positive group, and those negative for the 16 and 18/45 subtypes but positive for another high-risk subtype were classified as the other high-risk subtype positive group (Other HR-HPV+). See Fig. 1. for details.

## Method

### Liquid-based cytological examination

The exfoliated cells of the cervical canal and external orifice of the cervix were collected with a special brush designed for liquid-based cell tests (TCT). The specimens were made with a Liqun technology centrifuge and stained with Pap. Two professional pathologists made the cytological diagnosis. According to the TBS (2004) classification criteria, the TCT results were described as follows: no intraepithelial lesions or malignant lesions (NILM); atypical squamous cells-no clear meaning (ASCUS); low-grade squamous intraepithelial lesions (LSIL); atypical squamous cells without excluding high-grade intraepithelial lesions (ASC-H); high-grade squamous intraepithelial lesions (HSIL).

### Aptima HPV E6/E7 mRNA detection

Fourteen high-risk HPV E6/E7 mRNA types, including HPV 16, 18, 31, 33, 35, 39, 45, 51, 52, 56, 58, 59, 66 and 68, could be qualitatively detected by using the Hologic Panther automatic molecular detection platform. According to the specific steps of the Aptima HPV test kit (capture hybridization), the preservation solution of 1 ml cervical exfoliated cells was absorbed into the sample delivery medium (STM). Finally, the result was determined by the ratio of the signal to the threshold (s/C0). The HPV E6/E7 mRNA-positive cases were further typed and identified according to the Aptima HPV 16, 18/45 genotype detection kit (capture hybridization). This method could detect HPV 16 and HPV18/45 E6/E7 mRNA but could not distinguish between HPV18 and HPV45.

### Histopathological examination

A professional vaginoscope physician used an electronic colposcopy system (produced by Shenzhen Jinkewei Industrial Co., Ltd.) to selectively take multiple biopsies in the acetic acid white test and iodine test abnormal areas. If there was no obvious abnormality, cervical biopsies were performed at 3, 6, 9 and 12:00, and cervical canal curettage (ECC) was performed if necessary. The biopsy tissue was fixed and preserved in 4% neutral formalin, and the routine paraffin sections were diagnosed by 2 professional pathologists. The pathological grades included the following: (1) Changes in the normal or chronic inflammatory response; (2) Low-grade squamous intraepithelial lesion (LSIL); (3) High-grade squamous intraepithelial lesion (HSIL); (4) Invasive carcinoma of the cervix (Ca); LSIL and lower lesions referred to as LSIL-, HSIL and higher lesions referred to as HSIL+, including HSIL and invasive cancer.

### Statistical methods

The data were statistically analyzed by SPSS 26.0 software. The categorical data are expressed as the rate or percentage, the age is expressed as the mean ± standard deviation (x ± s), and the proportions in each group were compared by the chi-square test. The difference was statistically significant if P < 0.05.

## Results

### Distribution of different genotypes of HPV E6/E7 mRNA by age

**Fig. 2:**
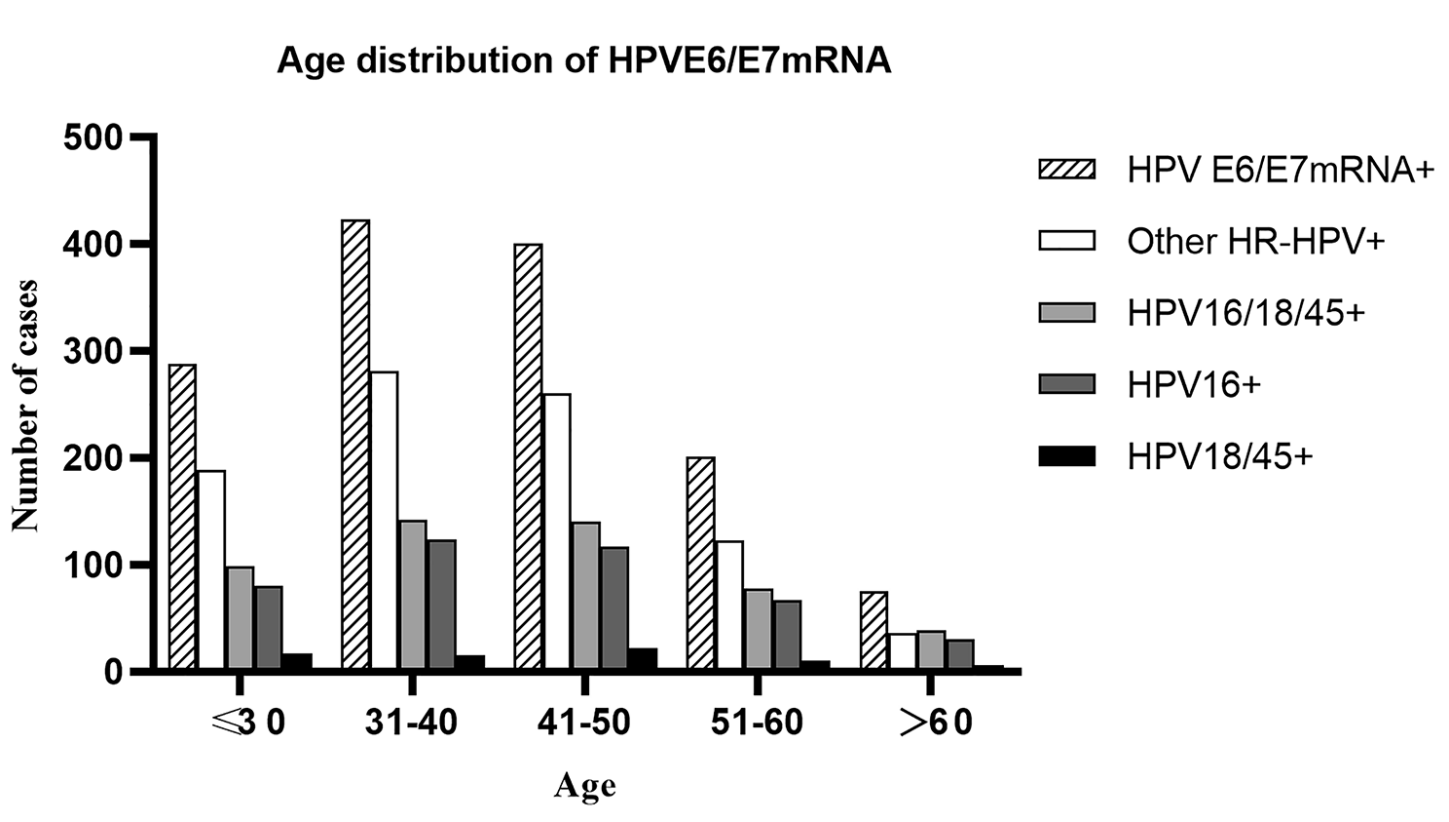
Age Distribution of HPV E6/E7 mRNA.

A total of 1387 cases were diagnosed as ASCUS and HPV E6/E7 mRNA positive by cytological screening. The positive rate for HPV16 was 30.1%, and the positive rate for HPV18/45 was 5.0%,The difference was statistically significant (P < 0.01). After grouping by age, the positive rate of HPV16 was significantly higher than HPV18/45 in all age groups. The positive rate of the HPV16/18/45 genotypes (35.9%) was significantly lower than that of the other HR-HPV genotypes (64.1%) (P < 0.01). Except for the > 60-year-old group, the positive rate of the HPV16/18/45 genotypes was significantly lower than that of the other HR-HPV types in the other age groups. The 31–40 years old group had the highest rate of HPV E6/E7+ (30.5%), followed by the 41–50 years old group (28.8%). The 31–40 years old group, followed by the 41–50 years old group, had the highest rate of HPV16+. The 41–50 years old group, followed by the ≤ 30 years old group, had the highest rate of HPV18/45+. See Fig. 2; Table 1 for details.

**Table 1:** The Distribution of Different HPV Genotypes at Different Ages, n (%)

	The total number	Age				
		≤ 30	31–40	41–50	51–60	＞60
HPV E6/E7 mRNA+	1387	288(20.8)	423(30.5)	400(28.8)	20(14.5)	75(5.4)
HPV16+	418(30.1)	80(5.8)	124(8.9)	117(8.4)	67(4.8)	30(2.2)
HPV18/45+	70(5.0)	17(1.2)	15(1.1)	22(1.6)	10(0.7)	6(0.4)
HPV16/18/45+	498(35.9)	99(7.1)	142(10.2)	140(10.1)	78(5.6)	39(2.8)
HPV16+/18/45+	10(0.7)	2(0.1)	3(0.2)	1(0.1)	1(0.1)	3(0.2)
Other-HRHPV+	889(64.1)	189(13.6)	281(20.3)	260(18.7)	123(8.9)	36(2.6)

### Comparative analysis of the positive results of HPV16/18/45 and other HR-HPV types and the pathological results of cervical biopsy

In the HPV16/18/45 + group, the biopsy results were normal in 137 cases (27.5%), which was significantly lower than that in the Other HR-HPV + group (48.4%), and the difference was statistically significant (P < 0.01); that is, the morbidity of HPV16/18/45 was significantly higher than that of the Other HR-HPV subtypes.

In the HPV16/18/45 + group, HSIL + was found in 219 cases (43.6%), which was significantly higher than that in the Other HR-HPV + group (15%), P < 0.01. That is, the morbidity of cervical HSIL + caused by subtypes of HPV16/18/45 was significantly higher than that of other subtypes of HR-HPV. See Table 2 for details.

**Table 2:** Comparison of Pathological Results Between the HPV16 + Group, HPV18/45 + Group, HPV16/18/45 + Group and the Other HR-HPV + Group, n (%)

		HPV 16+	HPV18/45+	χ^2^ value	HPV 16/18/45 +	Other hr-HPV+	χ^2^ value	P value
Pathological results of cervical biopsy	Normal	108(25.2)	31(38.8)	20.063	137(27.5)	430(48.4)	146.993	< 0.001
	LSIL	113(26.4)	34(42.5)		142(28.5)	326(36.7)		
	HSIL+	207(48.4)	15(18.8)		219(43.9)	133(15)		
		428	80		498	889		

### Comparative analysis of pathological results between HPV16-positive and HPV18/45-positive patients and cervical biopsy

The percentage of patients with normal biopsy results in the HPV16 + group (25.2%) was significantly lower than that in the HPV18/45 + group (38.8%). The difference was statistically significant (P < 0.01); that is, the morbidity of the HPV16 subtype was significantly higher than that of the HPV18/45 subtype. The proportion of HSIL + in the HPV16 + group (48.4%) was significantly higher than that in the HPV18/45 + group (18.8%) (P < 0.01). The morbidity of cervical HSIL + caused by the HPV16 subtype was significantly higher than that of the HPV18/45. See Table 2 for details.

### Menopausal stratification of the positive results of HPV16/18/45 and other HR-HPV compared with the pathological results of cervical biopsy

The proportion of HSIL + in the HPV16/18/45 group was 42.3% before menopause and 49.6% after menopause, and the difference was not statistically significant (P = 0.163). In the Other HR-HPV group, the proportion of HSIL + was 14.2% before menopause and 18.3% after menopause, and the difference was not statistically significant (P = 0.201). That is, there was no significant difference in the morbidity of HSIL + between premenopausal and postmenopausal patients caused by HPV16/18/45, and so was Other HR-HPV.

There were 17 cases of cervical cancer in the premenopausal HPV16/18/45 group (4.5%), which was significantly lower than the 26 cases of cervical cancer in the postmenopausal HPV16/18/45 group (22.2%) (P < 0.01). That is, the morbidity of cervical cancer in postmenopausal patients with HPV16/18/45 was significantly higher than that in premenopausal patients. Cervical cancer occurred in 12 cases (1.6%) in the premenopausal Other HR-HPV + group and 9 cases (5.7%) in the postmenopausal Other HR-HPV + group (P < 0.01); that is, the morbidity of cervical cancer in postmenopausal patients with Other HR-HPV was significantly higher than that in premenopausal patients. See Table 3 for details.

**Table 3:** Stratification According to Menopause: Comparison of Pathological Results Between HPV16/18/45 + and Other HR-HPV + Groups, n (%)

		Premenopausal group			Postmenopausal group		
			HPV16/18/45+ (%)	Other HR-HPV+ (%)		HPV16/18/45+ (%)	Other HR-HPV+ (%)
Pathological results of cervical biopsy	LSIL-	846(76.1)	220(57.7)	626(85.8)	189(68.5)	59(50.5)	130(81.8)
	HSIL+	265(23.8)	161(42.3)	104(14.2)	87(31.5)	58(49.6)	29(18.3)
	Ca	29(2.6)	17(4.5)	12(1.6)	35(12.7)	26(22.2)	9(5.7)
		1111	381(34.3)	730(65.7)	276	117(42.4)	159(57.6)
χ^2^ value			113.439			32.114	
P value			< 0.001			< 0.001	

### Menopausal stratification of the comparative analysis of the pathological results of cervical biopsy and positive HPV16 and HPV18/45

The proportion of HSIL + in the HPV16 group was 46.8% in the premenopausal group and 53.5% in the postmenopausal group (P = 0.241). There was no significant difference in HSIL + morbidity between premenopausal and postmenopausal patients with HPV16. The proportion of HSIL + in the premenopausal HPV18/45 group (9 cases, 15%) was significantly lower than that in the postmenopausal HPV18/45 group (6 cases, 30%), and the difference was statistically significant (P < 0.01). That is, the morbidity of HSIL + caused by HPV18/45 in postmenopausal patients was significantly higher than that in premenopausal patients. See Table 4 for details.

**Table 4:** Stratification According to Menopause: Comparison of Pathological Results Between HPV16 + and HPV16/18/45 + Groups, n (%)

		Premenopausal group		Postmenopausal group	
		HPV16+, n(%)	HPV18/45+, n(%)	HPV16+, n(%)	HPV18/45+, n(%)
Pathological results of cervical biopsy	LSIL-	174(53.3)	51(85.0)	47(46.5)	14(70.0)
	HSIL+	153(46.8)	9(15.0)	54(53.5)	6(30.0)
	Ca	17(5.2)	1(1.7)	24(23.8)	2(10.0)
		327	60	101	20
χ^2^ value		21.109		4.637	
P value		< 0.001		= 0.2	

Notes: Six cases were all positive for HPV16 and HPV18/45 in the premenopausal group, and 4 cases were all positive for HPV16 and HPV18/45 in the postmenopausal group

## Discussion

In recent years, the onset age of cervical cancer has tended to be younger, so early detection, early diagnosis and early treatment are more important for improving the prognosis of patients. Epidemiological data show that the highest incidence age of cervical cancer in situ is 30–35 years old and that of invasive cancer is 45–55 years old, and its incidence tends to be younger than in the past [14]. The age distribution curve of HPV infection is U-shaped [15]. The infection rate is the highest in the age group ≤ 25 years old, followed by the age group > 55 years old. In contrast, the results of this study showed that the highest proportion of HPV E6/E7 mRNA-positive patients was 31–40 years old, the second highest was 41–50 years old, and the lowest was > 60 years old, which may be due to the differences in HPV infection rate and subtype distribution among different regions and nationalities. All of the subjects in this study were ASCUS patients, which is different from most studies. Among them, HPV16 + was highest in the 31–40 years old group, followed by the 41–50 years old group, and for HPV18/45+, it was highest in the 41–50 years old group, followed by ≤ 30 years old.

It has been reported that most CIN2 lesions, especially in young women (< 30 years old), disappear spontaneously [16]. Therefore, the 31–50 years old group needs to be screened. The 2011 ACS, ASCCP, and ASCP joint guidelines and USPSTF guidelines recommend joint screening for women aged 30 to 65 [17], which is consistent with the results of this study. Patients over 50 years old will be further analyzed later in this article.

Ren et al. [18] found that the expression level of HR-HPV E6/E7 mRNA is a risk factor for high-grade cervical lesions and cervical cancer. However, the mechanisms of HSIL + and LSIL- are different. The mechanism of HSIL + is the persistent infection of HR-HPV, the integration of virus DNA into the human cell genome, and the persistently high expression of E6 and E7 in the host genome [19]. The mechanism of LSIL- is that low-risk HPV E6 and E7 proteins play a less important role in blocking the function of p53 and pRb than HR-HPVE6 and E7 proteins [20].

With regard to HR-HPV genotyping, some studies have shown that in patients with simple HPV positivity, the detection rate of HSIL + in HPV16/18-positive patients is significantly higher than that in other high-risk-positive patients [21], so the pathogenicity of HPV16 and HPV18/45 is worthy of further research and analysis. Consistent with this result, this study showed that the detection rate of HSIL + in the HPV16/18/45 group was 43.9%, significantly higher than that of the 15% in the Other HR-HPV + group. The detection rate of HSIL + in the HPV16 group was 48.4%, which was significantly higher than that of the 18.8% in the HPV18/45 + group; that is, the high-grade (HSIL+) morbidity of HPV16/18/45 to the cervix was significantly higher than that of the Other HR-HPV, while HPV16 was significantly higher than HPV18/45. Pruski et al. [22] found that high-grade squamous intraepithelial lesions were closely related to the positive expression of HPV E6/E7 mRNA. HPV-16 is the most common genotype in CIN2 + lesions diagnosed by LSIL in Chinese women, while HPV-18 is the most common genotype in CIN1 lesions [23], which is basically consistent with this conclusion. The results of this study also showed that the proportion of LSIL in the HPV18/45 + group (42.5%) was significantly higher than that in the HPV16 group (26.4%). Furthermore, it was concluded that HPV16/18/45 was more helpful than Other HR-HPV in screening for the pathogenesis of HSIL + in patients with ASCUS. Therefore, further colposcopy referral and biopsy diagnosis and treatment for HPV16/18/45-positive patients is necessary, but at the same time, Other HR-HPV still has 51.7% morbidity (including LSIL and the above lesions) and 15% high-grade morbidity. If this portion of ASCUS patients do not receive further examination, some HSIL or cervical cancer lesions will be ignored.

Moreover, undergoing colposcopy is not harmful, and the impression of colposcopy may help to increase the detection rate of high-level lesions compared with the psychological burden on patients. In cases of good communication by clinicians, the importance of avoiding missed diagnoses of high-level lesions is self-evident. It has been reported that for patients with simple HR-HPV infection, a treatment model based on risk stratification is feasible, and random biopsies are of little significance for patients with no abnormalities on colposcopy [24]. Therefore, we suggest that Other HR-HPV E6/E7 mRNA-positive ASCUS patients should also be referred for colposcopy according to the specific situation, and some biopsies can be performed according to the impression of colposcopy. Some studies suggest that unified referral for colposcopy examination should be considered for different subtypes of HPV E6/E7 mRNA-positive patients [25], which is consistent with the conclusions of this part.

In addition, the high proportion of ASCUS in the diagnosis of TCT is partly due to the difference between postmenopausal and premenopausal patients. In postmenopausal women, due to changes in hormone levels and cervical atrophy, the junction of the squamous epithelium and columnar epithelium returns to the cervical canal [26], and the lesions are difficult to detect. TCT cells in premenopausal women mainly come from the middle and surface layers, and the cervical cells of postmenopausal women generally atrophy, and thus their TCT cells mostly come from the bottom and sublayer cells. The ratio of nucleus to cytoplasm of these cells is slightly larger, which brings some difficulties to the diagnosis by cytologists. Due to changes in the physiological structure, the number of cells decreases in postmenopausal women, resulting in difficulties in TCT production and affecting the interpretation results [27].

Studies have shown that the percentage of HSIL detected by cervical curettage in postmenopausal patients (9.9%) is higher than that in premenopausal patients (2.6%) [28]. Combined with the HR-HPV test, it can provide better cervical cancer protection for postmenopausal patients than a simple TCT test [29]. Therefore, we compared the difference between premenopausal and postmenopausal patients with HPV E6/E7 mRNA detection. The incidence of HSIL + in postmenopausal patients was 31.5%, significantly higher than that in premenopausal patients (23.8%). The detection rate of HPV16/18/45 in premenopausal patients was 34.3%, which was significantly lower than that in postmenopausal patients (42.4%); that is, the HPV16/18/45 infection rate in postmenopausal patients was higher than that in premenopausal patients. However, some of the literature shows that [30] there is no significant difference in the positive rate of HPV between postmenopausal women and premenopausal women. The infection rate of high-risk HPV increases after 65 years of age, which is not contradictory to the results of this study because this study used HPV E6/E7 mRNA detection, which has higher specificity and sensitivity than simple HPV DNA detection in the previous literature. The object of this study is ASCUS patients, which is also different from the research subjects in most of the literature.

The percentage of HSIL + biopsies was 42.3% in the premenopausal HPV16/18/45 group and 49.6% in the postmenopausal HPV16/18/45 group; that is, there was no significant difference in the HSIL + morbidity of HPV16/18/45 between premenopausal and postmenopausal patients; at the same time, Other HR-HPV had no significant difference in HSIL + morbidity between premenopausal and postmenopausal patients. Then, the difference between premenopausal and postmenopausal morbidity caused by HPV16 and HPV18/45 was analyzed in detail: the proportion of HSIL + in the premenopausal HPV16 group was not significantly different from that in the postmenopausal HPV16 group. That is, there was no significant difference in high-grade morbidity between premenopausal and postmenopausal patients with HPV16. The proportion of HSIL + in the premenopausal HPV18/45 group was significantly lower than that in the postmenopausal HPV18/45 group, and the difference was statistically significant; that is, the high-grade morbidity of HPV18/45 in postmenopausal patients was significantly higher than that in premenopausal patients. Therefore, except for HPV18/45, there was no significant difference in the high-grade morbidity of HR-HPV between premenopausal and postmenopausal patients.

Combined with the above conclusions of this study, we can explain some of the reasons why the HSIL + incidence of postmenopausal patients is higher than that of premenopausal patients: (1) The high-grade morbidity of HPV16/18/45 was significantly higher than that of Other HR-HPV, (2) At the same time, the infection rate of HPV16/18/45 in postmenopausal patients was higher than that in premenopausal patients. (3) The high-grade morbidity of HPV18/45 in postmenopausal patients was significantly higher than that in premenopausal patients. Some studies have found that the older the age of acquiring an HPV infection, the higher the degree of cervical lesions [31], which is partly consistent with the conclusions of this study because we did not conduct a correlation analysis between the specific age and the degree of cervical lesions.

In the CastanonA literature [32], the incidence of cervical cancer in postmenopausal women showed an increasing trend. The results of this study showed that the incidence of cervical cancer in premenopausal women was 2.6%, which was significantly lower than that in postmenopausal women (12.7%), consistent with the above results. In addition, the proportion of premenopausal cervical cancer in the HPV16/18/45 group was significantly lower than that in postmenopausal women. That is, the pathogenicity of HPV16/18/45 in postmenopausal patients with cervical cancer is significantly stronger than that in premenopausal patients. These results also showed that the proportion of premenopausal cervical cancer in the Other HR-HPV group was significantly lower than that in the postmenopausal group; that is, the pathogenicity of Other HR-HPV in postmenopausal patients was also significantly stronger than that in premenopausal patients. Postmenopausal patients positive for Other HR-HPV should also be given attention. Therefore, the pathogenicity of HR-HPV to cervical cancer in postmenopausal patients is higher than that in premenopausal patients, which may be part of the reason why the incidence of postmenopausal cervical cancer is higher than that in premenopausal women.

It is mentioned in the literature that the incidence of cervical cancer in postmenopausal women is increasing, which may be due to the decrease in immune function in postmenopausal women [32], but it may not only be due to postmenopausal immunity. Combined with the results of this study: (1) The pathogenicity rate of the HPV16/18/45 subtype to HSIL + is significantly higher than that of Other HR-HPV. (2) The infection rate of HPV16/18/45 in postmenopausal patients was higher than that in premenopausal patients. (3) The pathogenicity rate of HPV18/45 to HSIL + in postmenopausal patients was significantly higher than that in premenopausal patients. (4) The pathogenicity rate of HR-HPV to cervical cancer in postmenopausal patients was higher than that in premenopausal patients. All of these factors may contribute to a significant increase in the incidence of cervical cancer in postmenopausal women.

Conclusions: In summary, this study suggests that colposcopy referrals and biopsy diagnosis and treatment should be performed for all HPV16/18/45 E6/E7 mRNA-positive and postmenopausal HR-HPV E6/E7 mRNA-positive ASCUS patients, and premenopausal ASCUS patients with other HR-HPV E6/E7 mRNA positivity should also be examined by colposcopy if possible according to the specific conditions to achieve early detection, early diagnosis and early treatment. However, while 16 and 18 of the high-risk HPV subtypes were reported separately in this study, no specific reports were made on the other subtypes. The pathological changes of the cervical epithelium may also need to consider other factors, such as the number of pregnancies and deliveries and sexual history, which need to be further analyzed to provide a more comprehensive and specific basis for further standardized clinical treatment of ASCUS patients, provide effective data for the accurate diagnosis of ASCUS in pathology, and make a modest contribution to the maximum improvement of the prognosis of patients.

## Data Availability

The datasets used and/or analysed during the current study available from the corresponding author on reasonable request.

## Abbreviations

ASC-US: atypical squamous cells of undetermined significance
NILM: negative for intraepithelial lesions or malignancies
HPV: human papillomavirus
HR-HPV: high-risk HPV
HPV16/18/45+: HPV16 + or HPV18/45+
HPV16+: HPV16 + and HPV18/45-
HPV18/45+: HPV16- and HPV18/45+
HPV16+/18/45+: HPV16 + and HPV 18/45+
LSIL: low-grade squamous intraepithelial lesion
HSIL: high-grade squamous intraepithelial lesion
